# Social Opportunity Produces Brain Changes in Fish

**DOI:** 10.1371/journal.pbio.0030390

**Published:** 2005-10-18

For many animals, possibly even for humans, mating success is determined by social status or dominance. A male's position in the “pecking order”—a term coined by the Norwegian Thorleif Schjelderup-Ebbe, who first proposed the concept of social dominance based on his work with chickens—can actually control his fertility. Social status also has other well-established, long-term physiological consequences. It can determine how big an animal grows, for example, or how it responds to stress. However, little is known about the neural mechanisms that link the social environment to the physiological changes associated with dominance.

Sabrina Burmeister, Erich Jarvis, and Russell Fernald are investigating these neural mechanisms in the cichlid fish Astatotilapia (Haplochromis) burtoni, in which dominance is tightly coupled to reproductive physiology. Subordinate males are less sexually mature, lack the bright blue or yellow body coloration and eye stripe that advertises dominance, and do not exhibit dominance behaviors such as territorial defense and courtship. Interestingly, the reproductive capacity of these male cichlids is socially regulated throughout life. A subordinate male can climb the cichlid social ladder if the dominant male in his social group dies as a result of predation or disease or if a subordinate stages a cichlid coup and dethrones the dominant male. The subordinate's sexual capacity then increases under the control of neurons in the preoptic area of the hypothalamus (a brain region that links the nervous system to hormonal systems), which enlarge and increase their expression of a peptide needed for reproduction called gonadotropin-releasing hormone 1 (GnRH1).

In their new research, Burmeister et al. have concentrated on the behavioral and genomic responses of subordinate male cichlids presented with an opportunity to ascend in status. The authors simulated natural social upheavals by removing the dominant male an hour before daylight (cichlids rely primarily on visual cues to monitor their social position) from a social group consisting of several females, a dominant male, and a subordinate male—an approach designed to represent naturally occurring behavioral and neural responses. The authors then watched the erstwhile subordinate male for behavioral signs of dominance, and measured changes in the expression of a class of genes called immediate-early genes within the brain. The proteins encoded by these genes—which show behavior-specific expression in many animals—induce the expression of other genes in the brain that produce changes in the animal's physiology. Subordinate and dominant males whose position in the social hierarchy had not been experimentally manipulated were similarly examined. [Fig pbio-0030390-g001]

**Figure pbio-0030390-g001:**
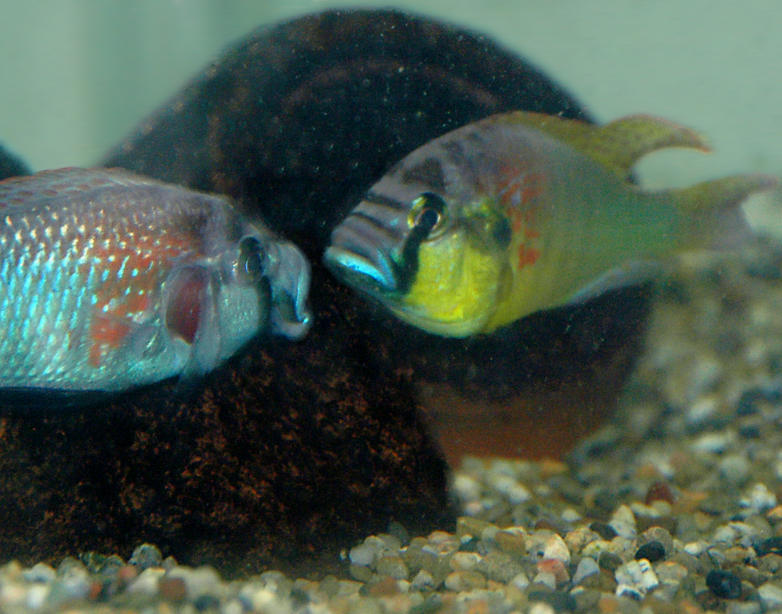
Dominant male Astatotilapia burtoni dispute a territorial boundary. (Image: Russell D. Fernald and Sabrina S. Burmeister)

The researchers discovered that subordinate males become dominant within minutes of an opportunity to do so, rapidly developing the bright coloration of dominant males and indulging in dominant behaviors. Over the same timescale, the expression of the immediate-early gene *egr-1* increased in the anterior preoptic region, peaking in those regions with the highest densities of *GnRH1*-expressing neurons. None of these changes occurred in unmanipulated, or stable, subordinate males. Furthermore, *egr-1* expression did not increase in stably dominant males, even though these fish showed similar behaviors to the ascending males, including the acquisition of dominance coloration, which is not maintained in the dark. In other words, the social opportunity itself rather than any behavior exhibited in response to the opportunity induced *egr-1* expression.

The *egr-1* gene encodes a transcription factor that is important for neural plasticity, and the *GnRH1* gene contains a binding site for egr-1. Thus, Burmeister et al. concluded, the social opportunity–induced expression of *egr-1* in the anterior preoptic area is an early trigger in a molecular cascade that ultimately produces the physiological changes associated with dominance in cichlids. Given that the preoptic region and GnRH are highly conserved in vertebrates, a similar neural mechanism could link social status to sexual physiology in other animals. —*Jane Bradbury*

