# Correction: Social Opportunity Produces Brain Changes in Fish

**DOI:** 10.1371/journal.pbio.3001429

**Published:** 2021-10-12

**Authors:** Jane Bradbury

The attribution of the image used in this article is incorrect. The correct attribution is: (Image: Henri Mouy).
